# Size-controlled and redox-responsive supramolecular nanoparticles

**DOI:** 10.3762/bjoc.11.260

**Published:** 2015-12-01

**Authors:** Raquel Mejia-Ariza, Gavin A Kronig, Jurriaan Huskens

**Affiliations:** 1 Molecular NanoFabrication group, MESA+ Institute for Nanotechnology University of Twente, P.O. Box 217, 7500 AE Enschede, The Netherlands

**Keywords:** host–guest interactions, nanoparticles, self-assembly, stimulus-responsive, supramolecular chemistry

## Abstract

Control over the assembly and disassembly of nanoparticles is pivotal for their use as drug delivery vehicles. Here, we aim to form supramolecular nanoparticles (SNPs) by combining advantages of the reversible assembly properties of SNPs using host–guest interactions and of a stimulus-responsive moiety. The SNPs are composed of a core of positively charged poly(ethylene imine) grafted with β-cyclodextrin (CD) and a positively charged ferrocene (Fc)-terminated poly(amidoamine) dendrimer, with a monovalent stabilizer at the surface. Fc was chosen for its loss of CD-binding properties when oxidizing it to the ferrocenium cation. The ionic strength was shown to play an important role in controlling the aggregate growth. The attractive supramolecular and repulsive electrostatic interactions constitute a balance of forces in this system at low ionic strengths. At higher ionic strengths, the increased charge screening led to a loss of electrostatic repulsion and therefore to faster aggregate growth. A Job plot showed that a 1:1 stoichiometry of host and guest moieties gave the most efficient aggregate growth. Different stabilizers were used to find the optimal stopper to limit the growth. A weaker guest moiety was shown to be less efficient in stabilizing the SNPs. Also steric repulsion is important for achieving SNP stability. SNPs of controlled particle size and good stability (up to seven days) were prepared by fine-tuning the ratio of multivalent and monovalent interactions. Finally, reversibility of the SNPs was confirmed by oxidizing the Fc guest moieties in the core of the SNPs.

## Introduction

Self-assembly and molecular recognition are two core concepts underlying supramolecular chemistry. These offer convenient and versatile pathways to nanostructured materials composed of molecular building blocks [1]. This fabrication strategy has been used to form supramolecular nanoparticles (SNPs) in which multiple copies of different building blocks interact via specific, non-covalent interactions [2]. They have the potential to be used in biomedical applications owing to control over their size, their assembly/disassembly, and the modular character for the versatile incorporation of agents aiming for imaging [3], photothermal therapy [4], drug delivery [5–7] and gene delivery [8–10] applications.

Different approaches have been used to form SNPs. Davis et al. showed the formation of SNPs using attractive electrostatic interactions between positively charged β-cyclodextrin (CD)-containing polymers and negatively charged siRNA at the core [8]. Neutral adamantyl-grafted poly(ethylene glycol) (Ad-PEG) was incorporated at the surface to stabilize these SNPs using host–guest interactions between CD and Ad. Tseng et al. studied the formation of SNPs that are assembled solely by host–guest interactions [11–12]. Here, the core is composed of multivalent interactions between positively charged CD-grafted polymers and positively charged poly(amidoamine) (PAMAM) dendrimers, and a monovalent neutral Ad-PEG stopper is introduced at the surface for stabilization. The SNP size was increased by increasing the amount of multivalent guest molecules in the core, while keeping the host and stopper concentration constant and having an excess of stopper to avoid precipitation. Wintgens et al. showed the formation of SNPs by controlling the host–guest ratio and the total concentration of components with a neutral polymer backbone [13]. Recently, our group [2,14–15] formed SNPs by varying the ratio of neutral monovalent stoppers and multivalent, positively charged guest dendrimer. Here, the overall concentration of the building blocks was kept constant while maintaining an equimolar stoichiometry of host and guest moieties. Moreover, our group [16] showed that SNP formation is controlled by a balance of forces between attractive supramolecular and repulsive electrostatic interactions using a multicomponent system based on a linear, negatively charged polymer. The force balance used in the latter approach was only observed with negatively charged polymers at low ionic strengths, and it is not known whether this balance occurs also for positively charged polymers and dendrimers.

In order to use these SNPs for biomedical applications, in particular for drug delivery, a stimulus-responsive self-assembled system is desired for controlled cargo release. Ferrocene (Fc) is a ubiquitous redox-responsive molecule that is associated with a reversible one-electron oxidation to the ferrocenium cation. At the same time, in its reduced state, Fc is a good guest for CD, but the affinity for CD is practically completely lost upon oxidation [17]. Thus, the formed CD-Fc inclusion complex disassembles when the Fc moiety is converted to the ferrocenium cation by electrochemistry [18] or by adding an oxidizing agent [19]. Different studies have employed this concept to form redox-responsive systems applied, for example, in self-healing materials [19], polymeric hydrogels [20–21], voltage-responsive vesicles [22], ultrasentive enzyme sensors [23], and as a plasma membrane protein isolation method [24]. So far, this concept has not been applied to SNPs.

Here, we aim to make SNPs with a redox-switchable assembly/disassembly mechanism. As a proof of concept, we used positively charged CD-grafted poly(ethylene imine) (CD-PEI) as a host, positively charged ferrocene-terminated PAMAM (Fc_8_-PAMAM) dendrimer as the multivalent guest and a monovalent stabilizer. Different stabilizers were used such as: Ad-PEG, Fc-terminated poly(ethylene glycol) (Fc-PEG), methoxypoly(ethylene glycol) (mPEG), and Ad-tetraethylene glycol (Ad-TEG). The effect of the following parameters on the formation of these SNPs is investigated: the role of ionic strength on SNP formation, the role of host–guest stoichiometry on the growth rate of the SNPs, and the influence of the affinity of the guest moiety and that of the PEG length of the stabilizer on the SNP stability. The size of the SNPs is controlled by the stoichiometry of the multivalent guest and the monovalent stabilizer. Finally, the reversibility of the SNPs is assessed by studying the influence of oxidation of the Fc moieties.

## Results and Discussion

### Characterization of building blocks

**Figure 1:**
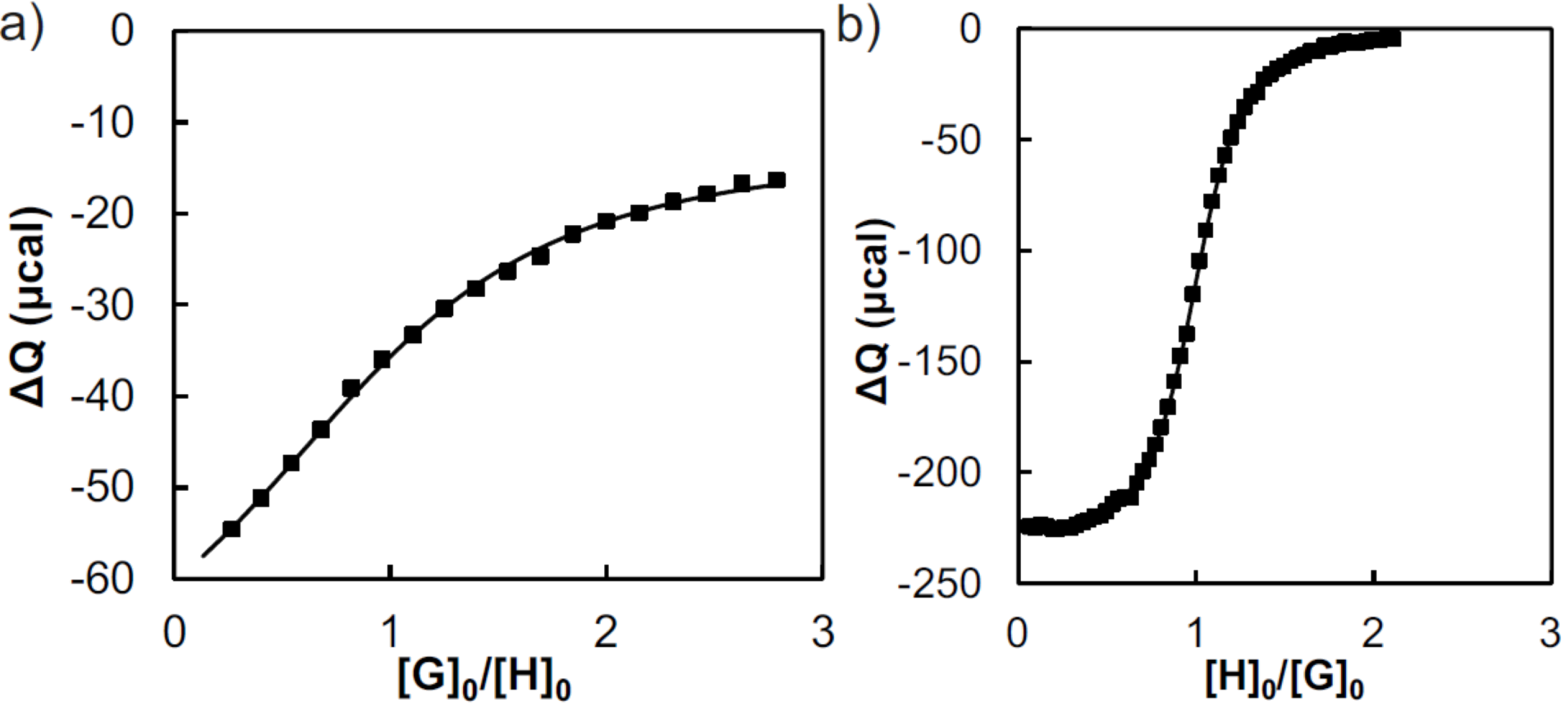
Microcalorimetric titrations of a) CD-PEI (CD concentration of 0.088 mM, cell) with Ad-TEG (1.1 mM, burette) and b) Ad-TEG (1.1 mM, cell) with CD (10 mM; burette). H = host (CD from CD-PEI or native CD) and G = guest (Ad from Ad-TEG). Experimental binding curve (markers) and best fit to a 1:1 model (line).

The positively charged host CD-PEI was synthesized according to earlier reports with slight modifications [25–26]. A reaction between 6-monotosyl-β-cyclodextrin (TsCD) and PEI in DMSO was performed using an excess of triethylamine as a base, followed by purification by dialysis. In order to control the stoichiometry of the host and guest moieties, the number of CDs per PEI was determined using microcalorimetry and NMR. According to the ^1^H NMR spectrum, the PEI backbone in the polymer building block CD-PEI is functionalized with, on average, 8 CD units. To assess the CD concentration in a CD-PEI stock solution, a calorimetric titration was performed using CD-PEI as the host and Ad-TEG as a guest, as shown in Figure 1a. Fitting the results by optimizing Δ*H*°, *K* and the CD host concentration gave a concentration of 0.39 mg/mL of CD-PEI, which is equivalent to a concentration of 0.088 mM of CD moieties participating in host–guest interactions. The results gave a binding constant (*K*_a_) of 3 × 10^4^ L mol^−1^. This is slightly lower than the interaction between native CD and Ad-TEG, for which a *K*_a_ value of 5 × 10^4^ L mol^−1^ has been determined (see Figure 1b). It can therefore be concluded that the grafting of CD to PEI has a minor effect on the host–guest binding affinity.

**Figure 2:**
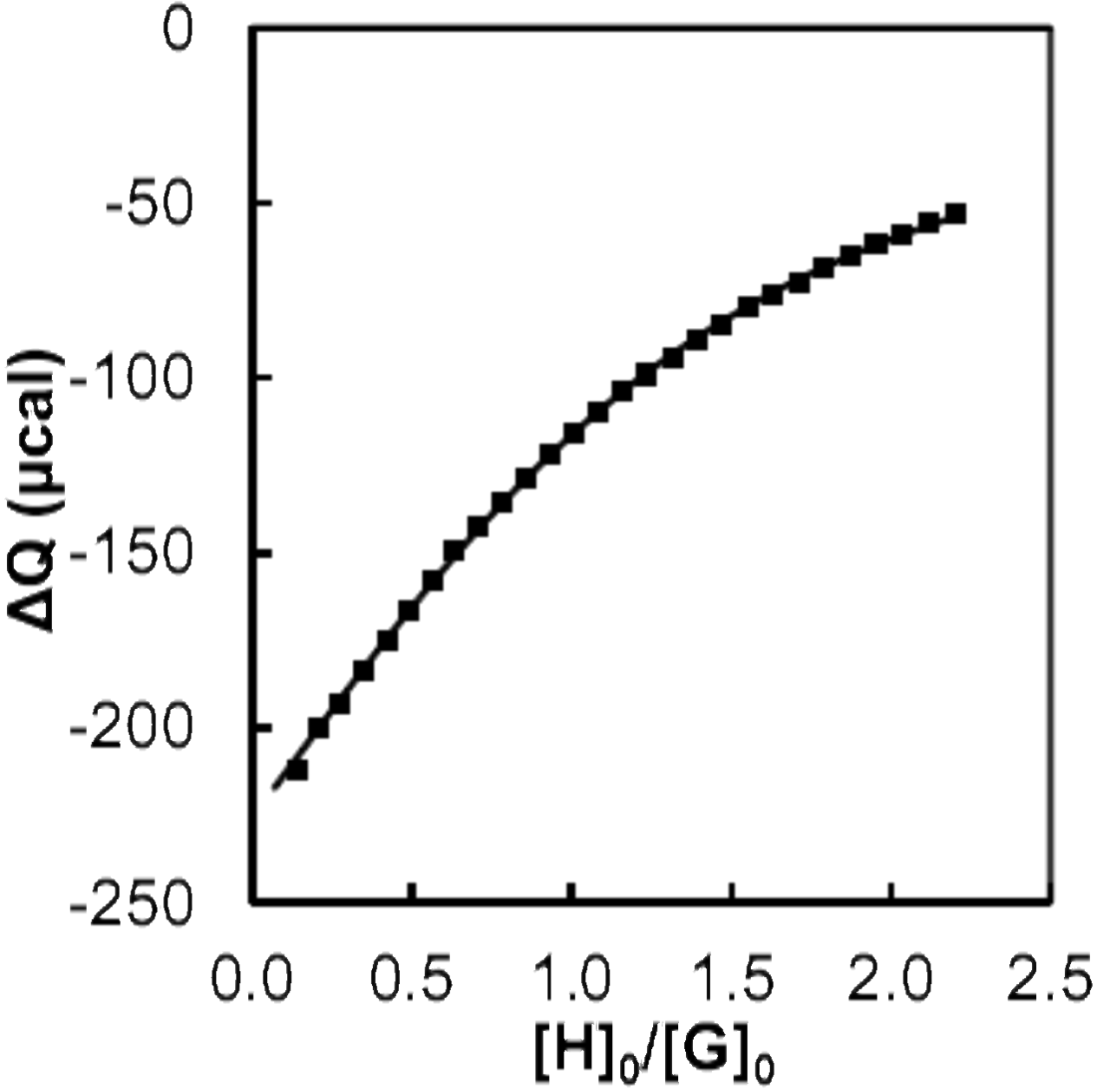
ITC titration of Fc-PEG (1.03 mM; cell) with native CD (10 mM; burette). H = host and G = guest. Experimental binding curve (markers) and best fit to a 1:1 model (line).

The positively charged Fc_8_-PAMAM multivalent guest was prepared according to a procedure developed in our group [27]. The positively charged Ad-terminated PAMAM (Ad_8_-PAMAM), used as a control, was prepared according to a literature procedure [11]. The neutral Fc-PEG stabilizer was synthesized by a reaction of 1-(chlorocarbonyl)ferrocene with the terminal amino group of methoxypoly(ethylene glycol)amine (*M*_w_ = 5000 g/mol) in dichloromethane, using an excess of triethylamine as a base, followed by precipitation from diethyl ether. To evaluate the association constant of the Fc moiety with free CD, and to confirm the degree of functionalization, a calorimetric titration was performed with native CD, as shown in Figure 2. This titration confirmed that nearly 100% of Fc-PEG was formed. The *K*_a_ of Fc-PEG with native CD is 1.2 × 10^3^ L mol^−1^, which is comparable to the binding constant of Fc dendrimers with CD [28].

The neutral stabilizer Ad-PEG was synthesized according to a literature procedure [11], by the reaction of 1-adamantylamine with the succinimidyl ester of carboxymethyl-PEG (*M*_w_ = 5000 g/mol) in dichloromethane with an excess of triethylamine.

### Formation and size control of SNPs

**Scheme 1:**
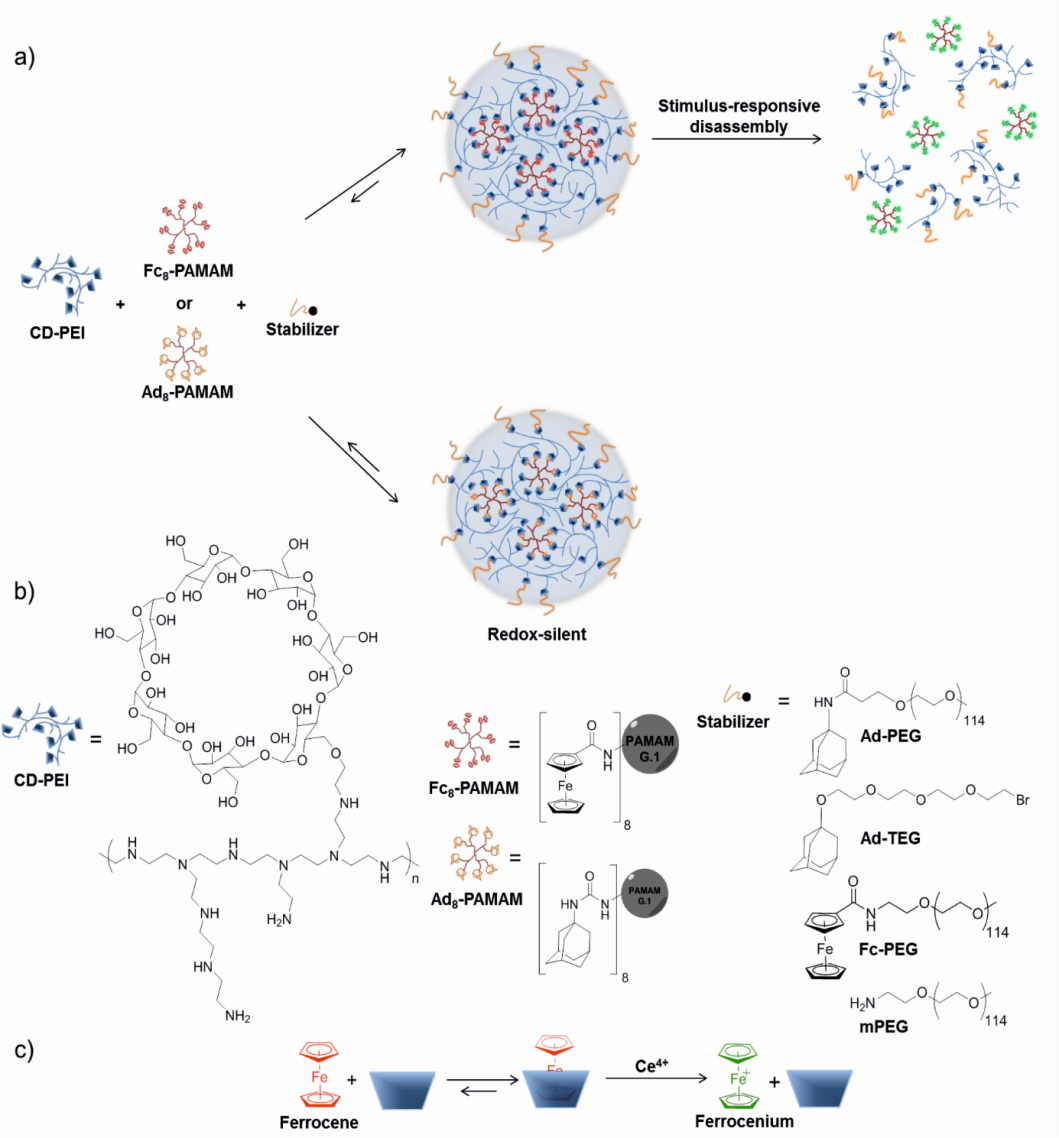
a) Schematic representation of the supramolecular nanoparticle (SNP) self-assembly and redox-triggered disassembly of the host–guest complex. b) Chemical structures of the building blocks used here. c) Binding of Fc by CD and subsequent dissociation upon oxidation of Fc.

Scheme 1a shows the concept of forming SNPs based on host–guest interactions, and the possible or impossible redox-induced disassembly when using Fc or Ad as the guest moiety, respectively. Throughout this study, concentrations of all the building blocks were expressed as the molar concentrations of the monovalent host and guest moieties, i.e., CD, Ad and Fc.

### Influence of the ionic strength

**Figure 3:**
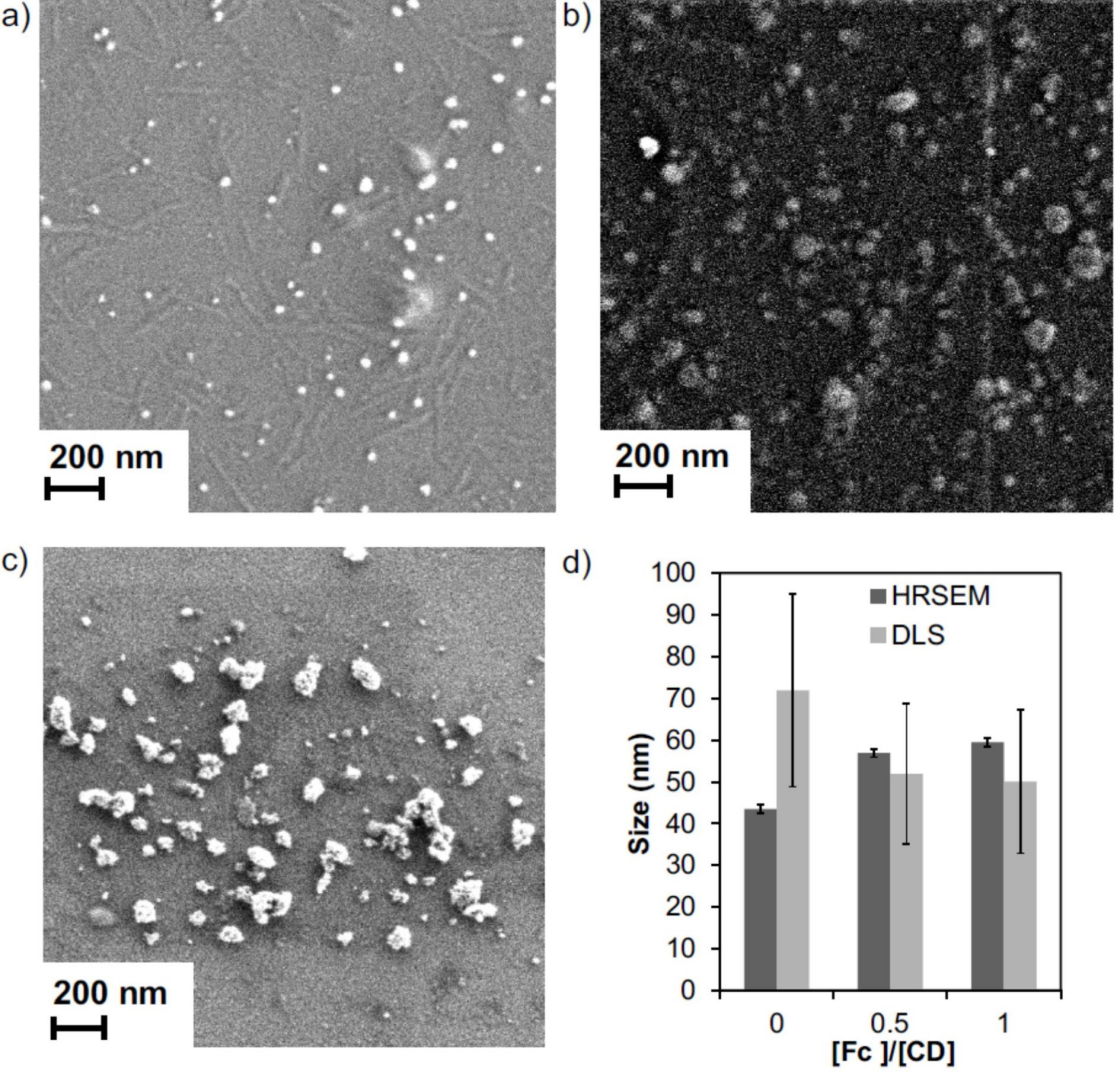
Size determination of SNPs prepared from CD-PEI and Fc_8_-PAMAM: SEM images (a–c) of the resulting SNPs as a function of the [Fc]/[CD] ratio (in Fc and CD moieties from Fc_8_-PAMAM and CD-PEI, respectively) in aqueous solution (without salt) (a: 0, b: 0.5 and c: 1) used during supramolecular assembly keeping constant the total concentration using [CD] = 100 µM and d) *d* by DLS and size by HRSEM.

The SNPs used here are formed using host–guest interactions between Fc_8_-PAMAM and CD-PEI. These molecules have positive charges that can influence the growth by repulsive interactions, which is an additional parameter that can influence the formation of SNPs. Moreover, Fc is used as the guest moiety as its stimulus-responsive properties lead to a triggered assembly/disassembly system. In order to study the influence of ionic strength on SNP formation, we used 0 to 0.2 M NaCl solutions and different guest–host ratios when assembling SNPs from CD-PEI and Fc_8_-PAMAM in the absence of stabilizer. SNPs were formed by adding Fc_8_-PAMAM (dissolved in DMSO) to an aqueous solution of CD-PEI ([CD] = 100 µM) in aqueous NaCl solution. To confirm particle formation, SNPs were characterized using DLS and SEM (Figure 3). DLS measurements of the particles in water without salt showed nanoparticles of comparable hydrodynamic diameters (*d*) for the different Fc/CD ratios. The size for 0% Fc (only CD-PEI) was approx. 70 nm, which is attributed to the fact that the concentration of CD-PEI is above its critical aggregation concentration. These results show that the particle size remains similar for the range of Fc/CD ratios shown here (in the absence of salt).

**Figure 4:**
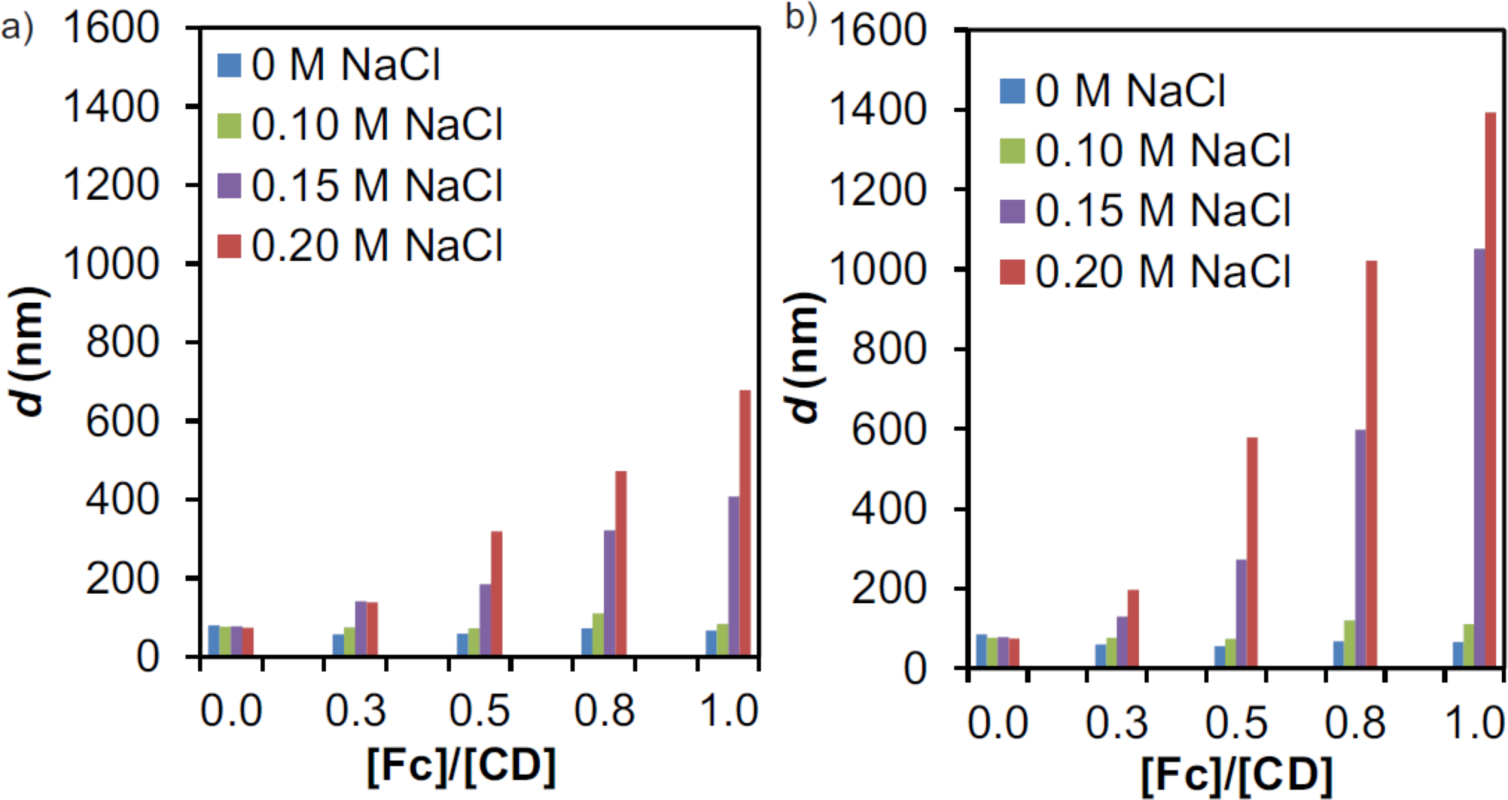
DLS size determination of SNPs prepared from CD-PEI and Fc_8_-PAMAM by increasing the [Fc]/[CD] ratio (in Fc and CD moieties from Fc_8_-PAMAM and CD-PEI, respectively) at different salt concentrations (0–0.2 M NaCl) keeping constant the total concentration using [CD] = 100 µM after: a) 20 min and b) 3 h.

Similar experiments were performed at three different salt concentrations, 0.1, 0.15 and 0.2 M NaCl, while keeping [CD] = 100 µM. Particle formation and growth was observed by DLS after 20 min and 3 h. Figure 4 shows an increase of particle size with increasing salt concentration at ionic strengths above 0.1 M, and the effect is stronger after 3 h, indicating a slow growth process. Up to an ionic strength of 0.1 M, however, no change of particle size was apparent.

Both host–guest and electrostatic interactions are at play here. Cyclodextrin host–guest interactions are largely hydrophobic in nature, and their affinity tends to increase slightly at increasing ionic strength. However, because of the already strong and multivalent nature [27–28] of the host–guest interactions at low ionic strengths, we do not expect such affinity differences to lead to the drastic stability differences observed here between the ionic strengths of 0.10 and 0.15 M. Regarding the electrostatic interactions, the Debye screening length is reduced to approx. 1 nm when increasing the ionic strength to 0.1 M. Moreover, zeta potential (ζ) measurements were performed using [CD] = 100 µM (CD is the number of moieties from CD-PEI) and [Fc] = 50 µM (Fc is the number of moieties from Fc_8_-PAMAM) at different salt concentrations after 20 min, as shown in Table 1. ζ decreased at increased ionic strengths, and values below 20 mV were observed at ionic strengths of 0.1 M and higher, indicating an absence of colloidal stabilization by charge repulsion at high ionic strength. These results demonstrate, as shown before for negatively charged polymers [16], that the aggregation is due to a loss of electrostatic colloidal stabilization. Thus, a balance between repulsive electrostatic forces and attractive host–guest interactions exists at low ionic strengths, leading to stable SNPs even in the absence of a stabilizer. At higher ionic strengths, however, the increased charge screening leads to a loss of electrostatic repulsion and therefore to aggregates that grow over time.

**Table 1:** Hydrodynamic diameters, *d*, and zeta potentials, ζ, measured by DLS of SNPs prepared at increasing salt concentrations (0–0.2 M NaCl) using CD-PEI and Fc_8_-PAMAM keeping the total concentration constant at [CD] = 100 µM and [Fc] = 50 µM (in Fc and CD moieties from Fc_8_-PAMAM and CD-PEI, respectively).

salt concentration (M)	*d* (nm)	ζ (mV)

0	51	33
0.1	58	17
0.15	247	15
0.20	441	15

### Influence of the host–guest stoichiometry on SNP formation

**Figure 5:**
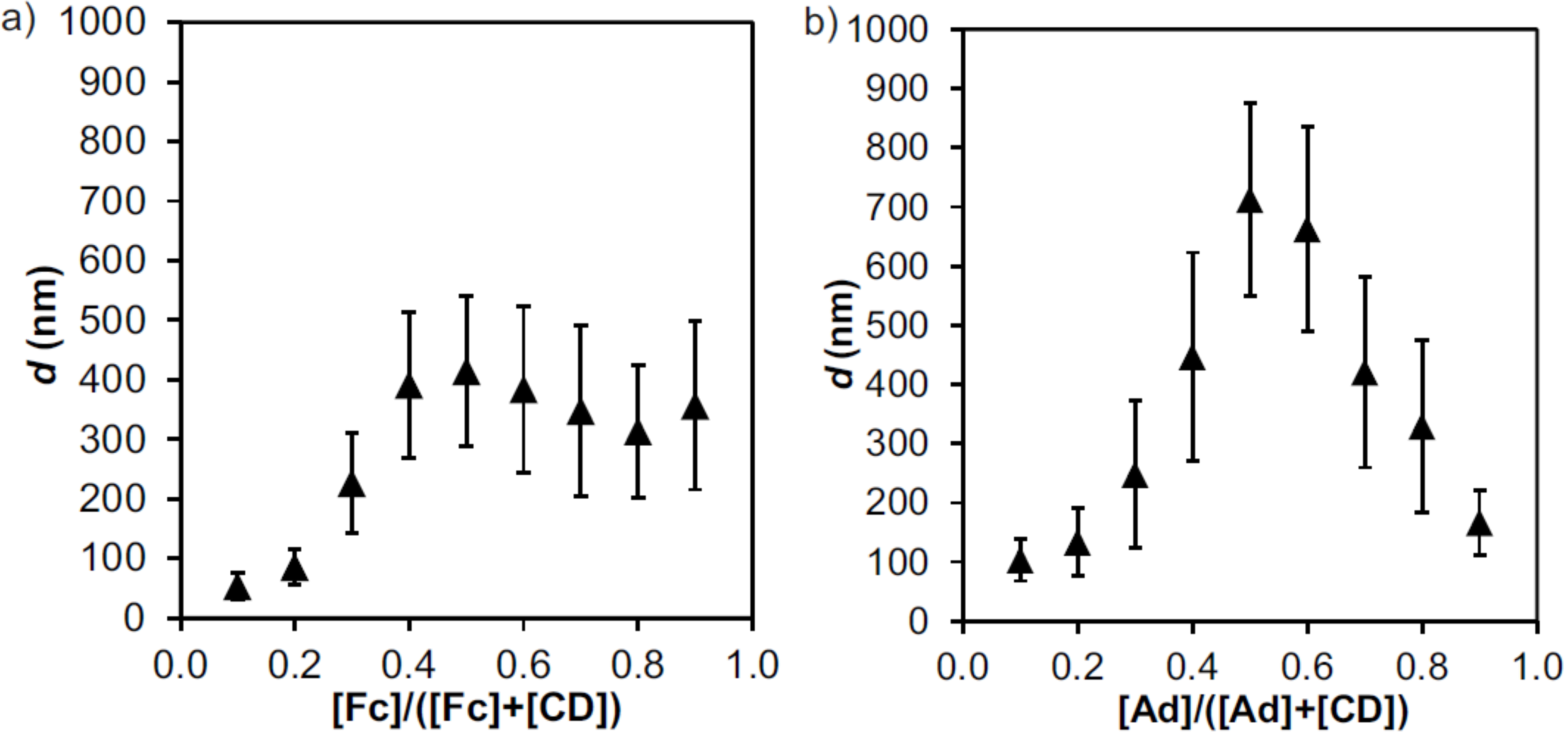
Hydrodynamic diameter, *d*, of SNPs prepared from CD-PEI and Fc_8_-PAMAM or Ad_8_-PAMAM measured by DLS as a function of the [guest]/([guest] + [CD]) ratio for: a) CD-PEI and Fc_8_-PAMAM [CD + Fc] = 50 µM (in CD and Fc moieties), *I* = 0.4 M NaCl, with 2 mM native CD measured after 10 min, and b) CD-PEI and Ad_8_-PAMAM [CD + Ad] = 200 µM (in CD and Ad moieties), *I* = 0.2 M NaCl, with 2.0 mM native CD measured after 6 min.

To assess whether all host and guest moieties of CD-PEI and Fc_8_-PAMAM are engaged in interactions, a Job plot was performed by varying the host–guest ratio while keeping the sum of the concentrations constant. The SNP growth at high ionic strength was used as a sign of interactions between the multivalent host and guest molecules. When increasing the Fc content to 0.5 (i.e., a host–guest ratio of 1:1), an increase in particle size was observed, but the particle size remained constant as the Fc concentration was increased further (data not shown). Therefore, 2 mM of native CD was added in an attempt to suppress non-specific, hydrophobically driven aggregation at excess Fc moieties. Figure 5a shows, however, a very similar picture, with particle sizes increasing as the Fc fraction was raised from 0 to 0.5, and a plateau of constant size at higher Fc fractions. Apparently, the addition of native CD was insufficient to cap excess free Fc groups, due to a lack of affinity. To verify that this low affinity is the main reason for the continued particle growth observed at Fc contents above 0.5, the Ad dendrimer analog was used as a control. Similar to the Fc case, increase of the Ad fraction up to 0.5 (see Figure 5b) led to an increase of the SNP size. Higher Ad contents in the presence of 2 mM native CD, however, led to a decrease in particle size, indicating that an excess of Ad is efficiently blocked by CD, which is in line with the approx. 30 times higher binding affinity of Ad (see above). Most importantly, this graph (Figure 5) confirms a 1:1 binding stoichiometry of the system. These results demonstrate that SNPs form optimally at a 1:1 stoichiometry at which all available host and guest moieties are simultaneously engaged in host–guest interactions.

### Effect of a monovalent stopper

**Figure 6:**
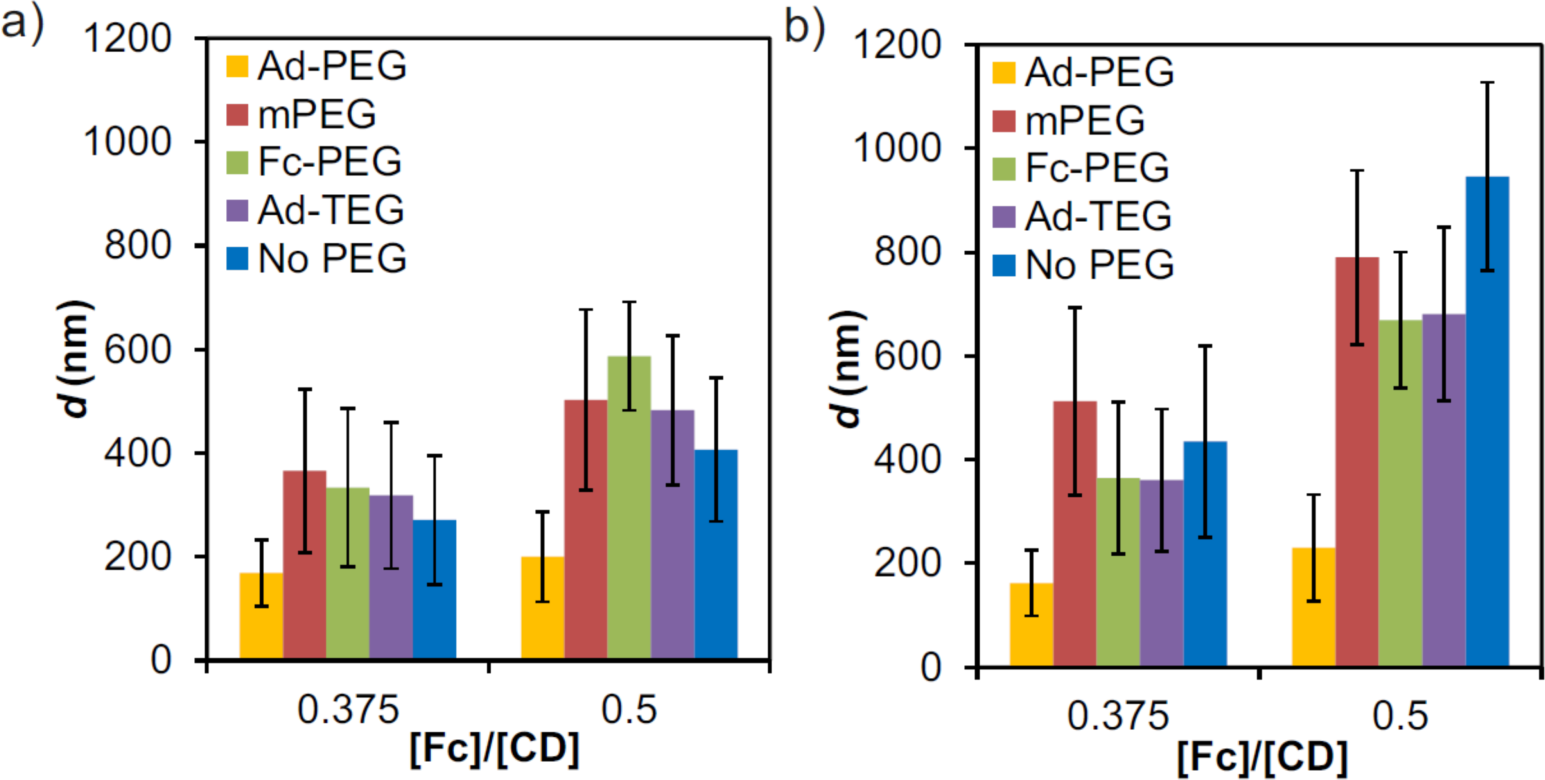
DLS size determinations of SNPs prepared from CD-PEI, Fc_8_-PAMAM, in the absence or presence of a monovalent stopper, for two [Fc]/[CD] ratios (in Fc and CD moieties from Fc_8_-PAMAM and CD-PEI, respectively) keeping constant both [CD] = [Fc] + [stopper] = 100 uM (where [stopper] is the concentration of the monovalent stopper), using 0.2 M NaCl and different stoppers: Ad-PEG, mPEG (no guest moiety), Fc-PEG, Ad-TEG and without stabilizer after: a) 20 min and b) 4 h.

In order to limit particle growth and achieve stabilization, a proper stabilizing agent should be found. The strategy on stoichiometry remains the same as previously described, keeping the host–guest ratio at 1:1 and a high ionic strength of 0.2 M NaCl. Two different parameters were considered to study the effect of a stopper: length and binding affinity. The formation of SNPs was studied using constant concentrations of [CD] = 100 µM (CD is the number of moieties from CD-PEI) and [Fc + guest-stabilizer] = 100 µM (Fc is the number of moieties from Fc_8_-PAMAM), thus keeping the molecular recognition moieties in a 1:1 stoichiometry ratio. For these experiments, first aqueous solutions of CD-PEI without or with a stabilizer (Ad-PEG, mPEG, Fc-PEG, Ad-TEG; see Scheme 1b) were prepared. Subsequently, Fc_8_-PAMAM (dissolved in DMSO) was injected into the respective aqueous solutions. Size tuning of the SNPs was assessed by using two different concentrations of Fc_8_-PAMAM dendrimers and stabilizer, while keeping the overall concentration of the guest moieties constant. The formation of SNPs was evaluated by DLS after 20 min and 4 h. Figure 6 shows the strong effect of the use of a stabilizer on the SNP size and, as shown before, that the SNP size is further increased by increasing the fraction of multivalent Fc moieties at the core of the particles. These results show that the smallest sizes and most stable particles were formed when using Ad-PEG as the stabilizer. Larger particles were observed for Fc-PEG than for Ad-PEG, but these also appeared stable (sizes after 20 min and 4 h are similar). The shorter Ad-TEG was less efficient in capping and stabilizing the SNPs compared to polymeric Ad-PEG. Leaving out the guest moiety, by using mPEG as a stabilizer, led to uncontrolled growth as was also observed in the absence of PEG. It should be noted that polymeric PEG derivatives have a critical aggregation constant that can be well below 1 µM [29]. We measured DLS for a 25 µM solution Ad-PEG and observed particles with a size of approx. 85 nm (data not shown), and others have observed sizes of 20–30 nm for different PEG derivatives [29]. However, the sizes reported here (Figure 6) for SNPs are much larger, most likely caused by larger abundance of SNPs compared to PEG aggregates and the higher response of SNPs by DLS. In particular the high similarity of the hydrodynamic sizes between the control (using the non-interacting mPEG) and the SNPs in the absence of stopper shows that the DLS data reported in Figure 6 are not convoluted by PEG aggregates. In summary, these results demonstrate that a guest moiety is important, and that a weaker guest is less efficient in stabilizing the particle. Moreover, steric repulsion by having a long polymer chain present on the stopper is important for achieving SNP stability.

### Size control by changing the stoichiometric composition

**Figure 7:**
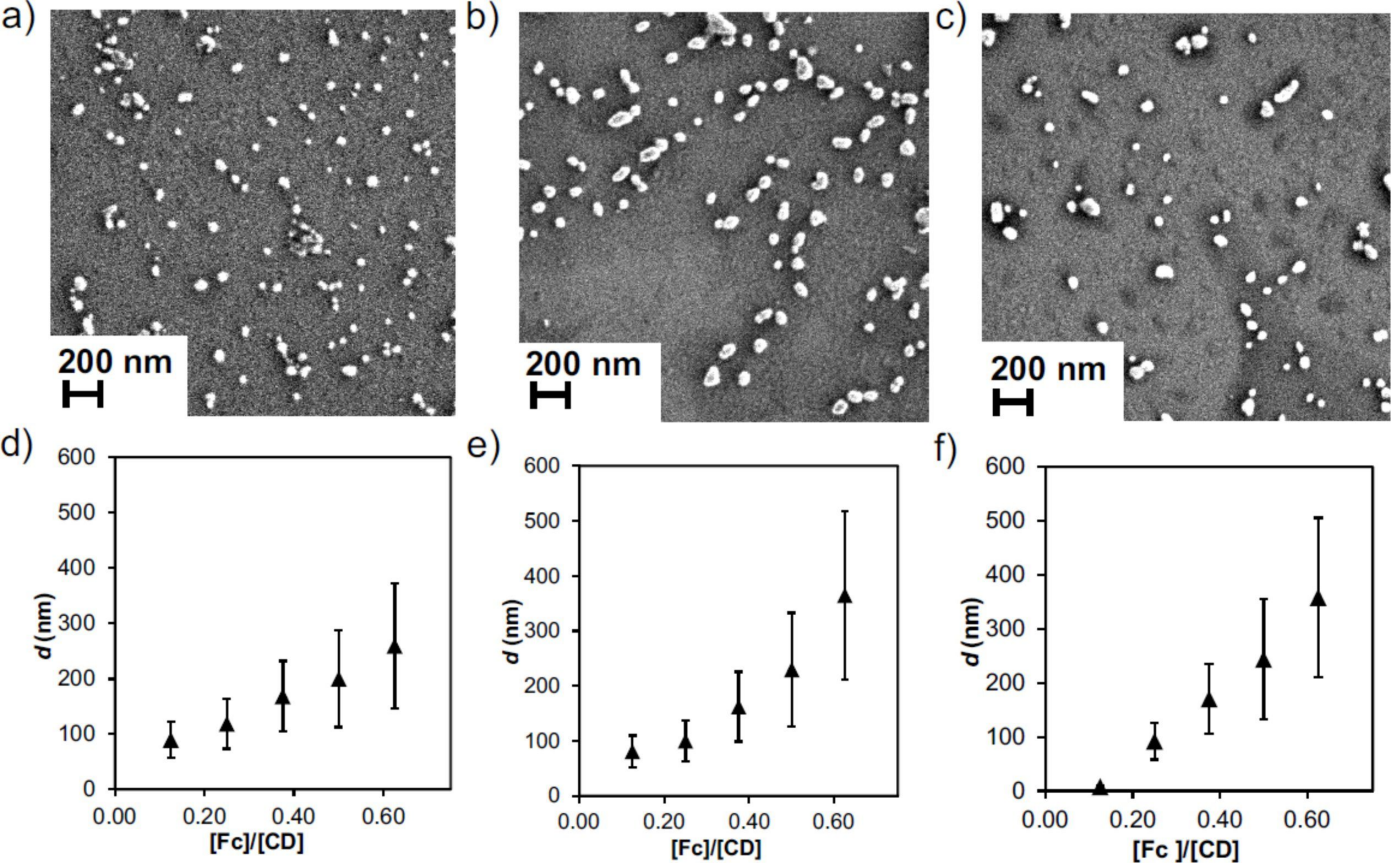
Size determinations of SNPs prepared from CD-PEI, Fc_8_-PAMAM and Ad-PEG: SEM images (a–c) of the resulting SNPs by increasing [Fc]/[CD] ratios (in Fc and CD moieties from Fc_8_-PAMAM and CD-PEI, respectively) using 0.2 M NaCl (a: 0.375, b: 0.50 and c: 0.625) used during supramolecular assembly using [CD] = 100 μM and CD:(Ad + Fc) stoichiometry, and DLS data (d–f) after: d) 20 min, e) 4 h and f) 7 days.

SNP size control was achieved by changing the stoichiometry of the multivalent guest and the monovalent stabilizer while keeping the overall host–guest ratio constant and equimolar. SNPs were observed by SEM and DLS for all samples as shown in Figure 7. The particle sizes determined by SEM (see Figure 7a–c) with sizes of 49 ± 13 nm (Fc fraction of 0.375), 61 ± 17 nm (Fc fraction of 0.5) and 67 ± 21 nm (Fc fraction of 0.625) were much smaller than those measured by DLS. This can be possibly due to drying effects. Figure 7d–f shows an increasing size with increasing fraction of the multivalent Fc_8_-PAMAM and they are stable up to 7 days. In summary, we have demonstrated the formation of stable and size-tunable SNPs by varying the multivalent vs monovalent stoichiometry.

### Stimulus-responsive disassembly by oxidation

**Figure 8:**
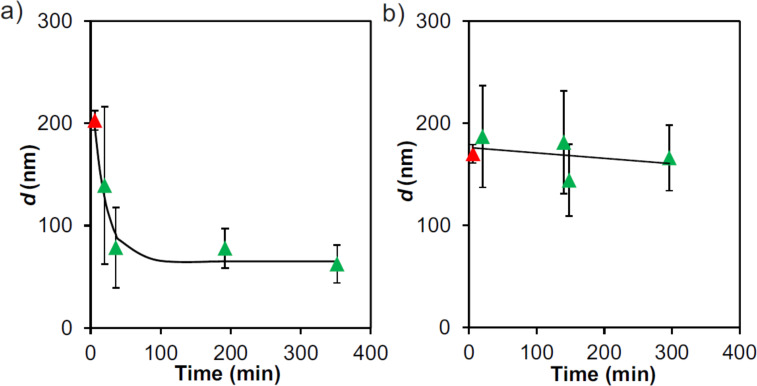
DLS size determination before (red) and after the addition of the oxidant agent Ce^4+^ (green) for as-prepared SNPs: a) [CD] = 100 µM (in CD moieties from CD-PEI) [Fc] = 50 µM (in Fc moieties from Fc_8_-PAMAM) and [Ad] = 50 µM (from Ad-PEG) and b) [CD] = 100 µM and [Ad] = 37.5 µM (in Ad moieties from Ad_8_-PAMAM) and [Ad] = 62.5 µM (from Ad-PEG) (control) in 0.2 M NaCl. 10 equiv of Ce^4+^relative to Fc was added to the SNPs. Experimental *d* measurements (markers) and trendlines (line, guide to the eye).

The redox-triggered disassembly of the Fc-containing SNPs (see Scheme 1a) makes use of the redox-responsive properties of Fc and the resulting loss of binding affinity for CD upon oxidation of Fc to the ferrocenium cation. We chose Ce^4+^ as the oxidizing agent to perform the disassembly experiments because of its proven effectiveness in a Fc/CD system similar to ours [30]. SNPs composed of CD-PEI, Fc_8_-PAMAM and Ad-PEG were formed using a ratio of CD/(Ad + Fc) = 1:1. The hydrodynamic diameter *d* by DLS was found to be 210 nm. Directly thereafter, a small volume of a Ce^4+^ stock solution was injected into the sample (Ce/Fc = 10). The SNP size was then monitored by DLS over time as shown in Figure 8a before (red) and after addition of Ce^4+^ (green) at 10 min. These results show a quick breakdown of the aggregates in the first 20 min. Sizes measured thereafter resemble the size measured for CD-PEI only. To prove that particle disassembly requires the redox-active Fc group, a similar experiment was performed using the redox-silent Ad_8_-PAMAM dendrimer (see Scheme 1a) as a control. SNPs composed of CD-PEI, Ad_8_-PAMAM and Ad-PEG were formed using [CD] = 100 µM (CD is the number of moieties from CD-PEI), [Ad] = 37.5 µM (Ad is the number of moieties coming from Ad_8_-PAMAM) and [Ad] = 62.5 µM (from Ad-PEG) in 0.2 M NaCl. A size of *d* ≈ 150 nm was measured by DLS. Figure 8b shows the hydrodynamic diameter of these aggregates over time before (red) and after addition of Ce^4+^ (green) at 10 min. These results shows that the Ad-based SNPs do not disassemble in the presence of oxidant. Therefore we conclude that Fc groups are needed to equip the SNPs with a triggered disassembly mechanism, attributed to the oxidation of the Fc groups of Fc_8_-PAMAM to the ferrocenium cation resulting in decomplexation of the guest groups and concomitant loss of multivalent links between the CD-PEI units in the SNPs.

## Conclusion

In conclusion, we have developed a strategy to form supramolecular nanoparticles using a redox-active host–guest complex as the interaction motif. The size of the resulting nanoparticles was controlled by different parameters, and SNP disassembly was achieved by using oxidation of the redox-active Fc moiety as the trigger. For the first time, we have shown that using positively charged building blocks, the size and stability of the supramolecular nanoparticles depend on a balance between repulsive electrostatic interactions between the charged building blocks and attractive host–guest interactions between the multivalent guest-functionalized dendrimers and host-functionalized polymers. At higher ionic strengths, the increased charge screening led to a loss of electrostatic repulsion and therefore to larger aggregates. Optimal self-assembly of the multivalent components was observed at a 1:1 stoichiometry of the host/guest moieties. A stabilizer with high binding affinity and sufficient steric repulsion is needed to obtain stable and small particles, thus Ad-PEG was observed to be the optimal stopper. Variation of the mono- to multivalent guest ratio provided a range of SNP sizes, and the SNPs were stable up to 7 days. The particles were successfully disassembled using a chemical oxidant. The understanding of the forces involved in SNP formation, and control over their stability and responsive character makes these SNPs a promising candidate for developing a drug delivery vehicle where control over the drug encapsulation and release can be achieved.

## Experimental

### Materials

Reagents and solvents were purchased from Sigma-Aldrich and used as received without further purification, unless noted otherwise. Millipore water with a resistivity of 18.2 MΩ cm at 25 °C was used in all the experiments. The amine-terminated poly(amido amine) dendrimer was purchased from Symo-Chem and received as a solution in methanol (20% w/w).

#### Synthetic procedures

The 6-monotosyl-β-cyclodextrin was synthesized according to a literature procedure [31]. The multivalent Fc_8_-PAMAM was prepared according to a procedure developed in our group [28]. Syntheses of Ad_8_-PAMAM and Ad-PEG (*M*_w_ = 5000 g/mol) were performed according to literature procedures [11], as well as Ad-TEG [32].

#### Synthesis of CD-PEI

The procedure for preparing the β-CD-functionalized PEI polymer was based on a literature procedure [11]. DMSO was freshly distilled under argon. Then, to a solution of branched poly(ethylene imine) [*M*_w_ ≈ 10,000 g/mol] (250 mg, 0.025 mmol) dissolved in 45 mL DMSO under argon at 60 °C, a solution of 6-monotosyl-β-cyclodextrin (1.4 g, 1.1 mmol) and 0.5 mL triethylamine in 35 mL of DMSO was added slowly under argon by using a syringe while stirring. The resulting solution was stirred at 60 °C for three days under argon. The solution was cooled to room temperature and diluted with 40 mL deionized water with a resulting pH of 10.9. The solution was transferred to a Spectra/Por MWCO 6–8 kD membrane and dialyzed against water for 4 days. The dialyzed solutions were filtered over paper and lyophilized to afford 189 mg of a fluffy, near-white solid. ^1^H NMR (400 MHz, D_2_O) δ (ppm) 5.39–5.05 (br, 7H, C1H of CD), 3.87–3.56 (m, 42.1, C2-6H of CD), 3.5–2.2 (br, 115.6, OCH_2_ of PEI).

#### Synthesis of Fc-PEG

Methoxypoly(ethylene glycol)amine (*M*_w_ = 5000 g/mol; 250 mg, 0.050 mmol) and 0.2 mL triethylamine was dissolved in 15 mL CH_2_Cl_2_ under argon in a 100 mL one-necked round-bottom flask. While stirring, a solution of ferrocenoyl chloride (500 mg, 2.0 mmol) in 15 mL dichloromethane was added dropwise by using a syringe. The mixture was allowed to stir overnight at room temperature under argon. The solvent was removed leaving an orange residue. The residue was dissolved in 20 mL chloroform. The chloroform mixture was washed with 10 mL aqueous saturated NaHCO_3_ solution after which the organic layer was dried using MgSO_4_. After filtration over paper, the solvent was removed by evaporation and the remaining precipitate was redissolved in 2 mL chloroform. The chloroform solution was added dropwise to 40 mL of diethyl ether, giving immediate precipitation of a yellow solid, which was filtrated and dried in a vacuum oven at 40 °C overnight. This yielded 136 mg of a slightly yellow solid. ^1^H NMR (400 MHz, D_2_O) δ (ppm) 4.78 (m, 2H, Fc), 4.53 (t, 2H, Fc), 4.28 (s, 5H, Fc), 3.85 (t, 2H, CH_2_CH_2_NHCO), 3.50 (t, 2H, CH_2_NHCO), 3.36 (s, 3H, OCH_3_).

### Methods

#### Supramolecular nanoparticle assembly as a function of ionic strength

For the preparation of SNPs as a function of ionic strength (0–0.2 M NaCl), aqueous solutions of CD-PEI and NaCl and Fc_8_-PAMAM in DMSO were prepared before mixing. The concentration of CD-PEI was kept constant. As an example, for preparing a solution of 50% Fc entities derived from the Fc dendrimer in 0.1 M NaCl, first 100 µL of aqueous CD-PEI solution (500 µM in CD moieties), 60 µL of aqueous NaCl solution (833.3 mM) and 340 µL of water were mixed for 30 s. After mixing, 7.5 µL of Fc_8_-PAMAM solution in DMSO (3336 µM in Fc moieties) was injected to the previous solution while sonicating.

#### Job plot using Fc_8_-PAMAM

For the preparation of SNPs using 0.4 M NaCl, an aqueous solution of CD-PEI, free CD, NaCl and a solution of Fc_8_-PAMAM in DMSO were prepared before mixing. The concentration of total moieties was kept constant at 50 µM. As an example, for preparing a solution of 50% Fc entities (25 µM) derived from the Fc dendrimer, first 125 µL of aqueous CD-PEI/free CD solution (100 µM in CD moieties; 2 mM free CD), 240 µL of aqueous NaCl/free CD solution (833.3 mM NaCl; 2 mM free CD) and 135 µL of aqueous free CD solution (2 mM), were mixed for 30 s. After mixing, 7.5 µL of Fc_8_-PAMAM solution in DMSO (1664 µM in Fc moieties) was injected to the previous solution while sonicating.

#### Job plot using Ad_8_-PAMAM

For the preparation of SNPs using 0.2 M NaCl, aqueous solution of CD-PEI, free CD, NaCl and solution of Ad_8_-PAMAM in DMSO were prepared before mixing. The concentration of total moieties was kept constant at 200 µM. As an example, for preparing a solution of 50% Ad entities (100 µM) derived from the Ad dendrimer, first 125 µL of aqueous CD-PEI/free CD solution (400 µM in CD moieties; 2 mM free CD), 120 µL of aqueous NaCl/free CD solution (833.3 mM NaCl; 2 mM free CD) and 255 µL of aqueous free CD solution (2 mM), were mixed for 30 s. After mixing, 7.5 µL of Ad_8_-PAMAM solution in DMSO (6664 µM in Ad moieties) was injected to the previous solution while sonicating.

#### Supramolecular nanoparticle assembly as a function of different stoppers

For the preparation of SNPs using 0.2 M NaCl as a function of stoppers at two different Fc fractions, various aqueous solutions of CD-PEI, PEG modified (Ad-TEG, Ad-PEG (*M*_w_ = 5000 g/mol), Fc-PEG (*M*_w_ = 5000 g/mol), mPEG (*M*_w_ = 5000 g/mol) and using two different concentrations of Fc_8_-PAMAM in DMSO were prepared. The concentration of CD-PEI was kept the same. As an example, for preparing a solution of 50% Fc entities derived from the Fc dendrimer using Ad-PEG, first 100 µL of aqueous CD-PEI solution (500 µM in CD moieties), 100 µL of aqueous Ad-PEG solution (250 µM), 120 µL of aqueous NaCl solution (833.3 mM) and 180 µL of DI water were mixed for 30 s. After mixing, 7.5 µL of Fc_8_-PAMAM solution in DMSO (3336 µM in Fc moieties) was injected to the previous solution while sonicating.

#### Supramolecular nanoparticle assembly as a function of increasing multivalent guest

For the preparation of SNPs in 0.2 M NaCl various aqueous solution of CD-PEI and Ad-PEG and Fc_8_-PAMAM in DMSO were prepared before mixing. The concentration of CD-PEI was kept the same. As an example, preparing a solution of 50% Fc entities derived from the Fc dendrimer, first 100 µL of aqueous CD-PEI solution (500 µM in CD moieties), 100 µL of aqueous Ad-PEG solution (250 µM), 120 µL of aqueous NaCl solution (833.3 mM) and 180 µL of DI-water were mixed for 30 s. After mixing, 7.5 µL of Fc_8_-PAMAM solution in DMSO (3336 µM in Fc moieties) was injected to the previous solution while sonicating.

#### Triggered disassembly of SNPs

To evaluate the redox responsiveness of the particles, SNPs containing [CD] = 100 µM (in CD moieties from CD-PEI), [Fc] = 50 µM (in Fc from Fc_8_-PAMAM) and [Ad] = 50 µM (from Ad-PEG) were prepared in 0.4 M NaCl solution. The *d* was measured over time after the injection of Ce^4+^ (10 equiv of Ce^4+^ relative to Fc was added to the SNPs). To evaluate whether the SNP disassembly was due to the oxidation of the ferrocene groups, SNPs containing [CD] = 100 µM (in CD moieties from CD-PEI), [Ad] = 37.5 µM (in Ad from Ad_8_-PAMAM) and [Ad] = 62.5 µM (from Ad-PEG) were prepared in 0.2 M NaCl. The *d* was measured over time after the injection of Ce^4+^ (10 equiv of Ce^4+^ relative to Ad was added to the SNPs).

### Equipment

#### Dynamic light scattering (DLS)

Hydrodynamic diameters and zeta potentials were measured on a Zetasizer NanoZS (Malvern Instrument Ltd, Malvern, United Kingdom) at 20 °C, with a laser wavelength of 633 nm and a scattering angle of 173°.

#### High resolution scanning electron microscopy (HR-SEM)

All SEM images were taken with a Carl Zeiss Merlin scanning electron microscope. The samples were prepared by drop-casting 10 μL of a SNP solution onto a silicon wafer. After 60 s, excess of water was removed by filter paper. The particle dimensions are obtained from SEM images with ImageJ software. For each sample at least 100 particles were measured.

#### Calorimetric analysis

Calorimetric titrations were performed at 25 °C using a Microcal VP-ITC titration microcalorimeter. Sample solutions were prepared in Millipore water.

#### NMR spectroscopy

^1^H NMR spectra was recorded on a Bruker 400 MHz NMR spectrometer. ^1^H chemical shift value, 400 MHz is reported as δ using the residual solvent signal as internal standard at ≈22 °C.

#### Mass spectrometry

Mass analysis was done using matrix-assisted laser desorption ionization (MALDI) on a Waters Synapt G1 using 2,5-dihydroxybenzoic acid as the matrix.
